# Supplementary material for: Juglone Suppresses LPS-induced Inflammatory Responses and NLRP3 Activation in Macrophages
Source: Molecules. 2020 Jul 7;25(13):3104. doi: 10.3390/molecules25133104 (PMC7412499; doi:10.3390/molecules25133104)

Supplementary Materials:

Figure S1: Original images for Western blots of IL-1β, IL-18 and β-actin.

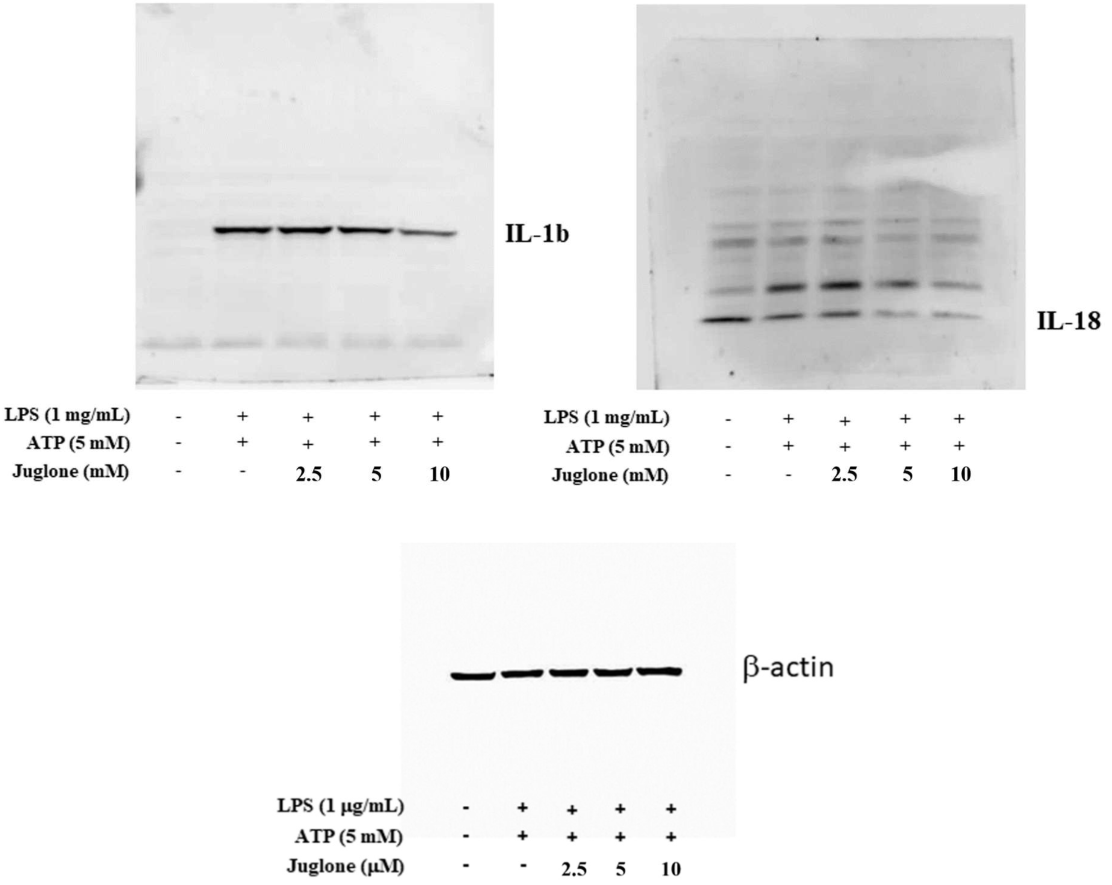

Figure S2: Original images for Western blots of NLRP3, pro-caspase and cleaved caspase-1.

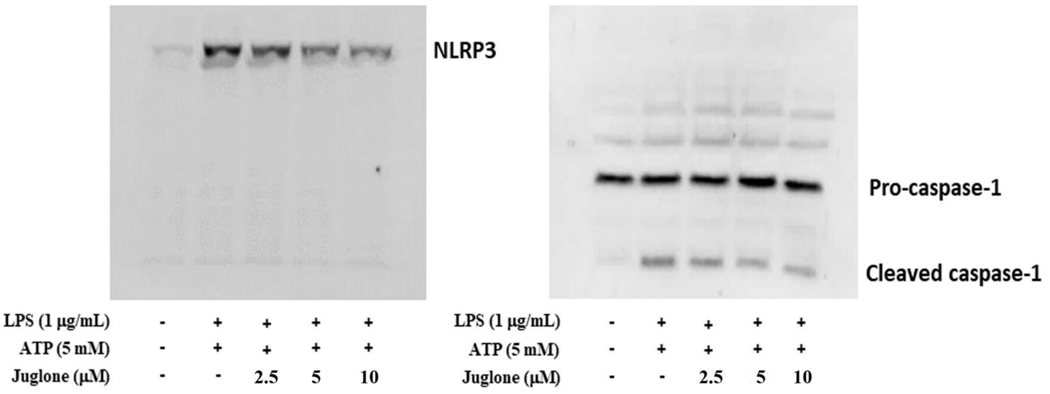

Supplement: Supplementary file 1 [file molecules-25-03104-s001.pdf]
